# Effect of a Music Therapy Intervention Using Gerdner and Colleagues’ Protocol for Caregivers and Elderly Patients with Dementia: A Single-Blind Randomized Controlled Study

**DOI:** 10.3390/jpm11060455

**Published:** 2021-05-23

**Authors:** Guido Edoardo D’Aniello, Davide Maria Cammisuli, Alice Cattaneo, Gian Mauro Manzoni, Enrico Molinari, Gianluca Castelnuovo

**Affiliations:** 1 Istituto Auxologico Italiano IRCCS, Psychology Research Laboratory, 20122 Milan, Italy; al.cattaneo@auxologico.it (A.C.); gianmauro.manzoni@uniecampus.it (G.M.M.); molinari@auxologico.it (E.M.); gianluca.castelnuovo@unicatt.it (G.C.); 2 Department of Psychology, Catholic University of the Sacred Heart, 20123 Milan, Italy; dm.cammisuli@gmail.com; 3 Faculty of Psychology, eCampus University, 20060 Novedrate, Italy

**Keywords:** music therapy, dementia, caregiver, RCT

## Abstract

Music therapy (MT) is considered one of the complementary strategies to pharmacological treatment for behavioral and psychological symptoms (BPSD) of dementia. However, studies adopting MT protocols tailored for institutionalized people with dementia are limited and their usefulness for supporting caregivers is under investigated to date. Our study aimed at evaluating the effects of an MT intervention according to Gerdner and colleagues’ protocol in a sample of 60 elderly people with moderate-to-severe dementia of the Auxologico Institute (Milan, Italy) and associated caregivers, randomly assigned to an Experimental Group (EG) (*n* = 30) undergoing 30 min of MT two times a week for 8 weeks and to a Control Group (*n* = 30) (CG) receiving standard care. Before and after the intervention, residents-associated caregivers were administered the Caregiver Burden Inventory (CBI) and the Neuropsychiatric Inventory (NPI). Depression and worry were also assessed in caregivers prior to the intervention, by the Beck Depression Inventory-II and the Penn State Worry Questionnaire, respectively. A mixed model ANCOVA revealed a Time*Group effect (*p* = 0.006) with regard to CBI decreasing after the intervention for the EG and Time*Group effects (*p* = 0.001) with regard to NPI_frequencyXseverity and NPI_distress, with a greater effect for the EG than the CG. Implications for MT protocols implementations are discussed.

## 1. Introduction

Behavioral and psychological symptoms of dementia (BPSD) refer to the spectrum of non-cognitive and non-neurological features significantly impacting on prognosis and patient management and constitute a major component of the disease, irrespective of its subtypes [1]. As dementia is a progressive disease, BPSD worsen over time, requiring higher support and increased sanitary and care costs [2]. The BPSD improve caregivers’ burden and distress [3] and are related to an increased level of dependence according to the progression of the disease [4]. Indeed, many studies have focused on the stressors associated with caregivers’ support. Remarkably, caregivers’ coping strategies and personality factors seem to play a critical role towards controlling BPSD [5]. Further, BPSD increasing causes higher caregiver distress [3].

It has been estimated that the prevalence of BPSD in people with dementia living in institutional settings is approximately 91–96% [1] and the majority of patients mainly present with an outcome of neuropsychiatric symptoms such as depression, apathy, irritability, anxiety, euphoria, hallucination and disinhibition [6]. One of the most extensively used instruments to assess BPSD is the Neuropsychiatric Inventory (NPI) [7]. Validity and reliability of the NPI have been established in different languages; it can evaluate 12 symptoms based on a caregiver’s interview about patient (i.e., delusions, hallucinations, agitation, depression, anxiety, apathy, irritability, euphoria, disinhibition, aberrant motor behavior, night-time behavior disturbances, and eating behavior abnormalities) covering a wide range of symptoms associated with progressive dementia states. Treatment of BPSD currently represents a relevant therapeutic challenge for patients with moderate-to-severe dementia because of their difficulty in explaining feeling and emotions and agitation reported in the course of the disease [8]. Particularly, people with moderate-to-severe dementia are at higher risk of developing aggression [9], in terms of violent behavior and physically/verbally inappropriate responses to environmental stimuli [10].

Pharmacological treatment usually constitutes the primary approach to excessive behaviors but adverse effects of medication (e.g., speech inhibition, diminished language skills, altered gait and falls, and even a more severe cognitive deterioration) may occur in the treatment course [11], with negative consequences on patients’ global status. Non-specific experiences such as music listening, touch therapy, and hand massage may be beneficial for calming neuropsychiatric symptoms presented by patients with moderate-to-severe dementia [12]. Specifically, Music Therapy (MT) represents a non-pharmacological complementary strategy to pharmacological treatment for dealing with neuropsychiatric symptoms of people with dementia [13]. Recent advancements improving personalized medicine in research, diagnosis and treatment of dementia have sustained a more comprehensive approach for patients, with the aim of better finalizing scientific knowledge to tailored interventions starting from data integration about an individual’s specific pattern of genetic variability, environment and lifestyle factors [14].

Through non-verbal behavior and sound-music performances, MT allows participants to convey their emotions and feelings, establish a contact with significant others and modify their affective status and interpersonal communication, with a positive adaptation to their social environment. In particular, Gerdner and colleagues’ protocol [15,16] supports the fact that archaic expressive and relational non-verbal abilities persist across a person’s life span and may be reactivated by MT as interpersonal modalities of relationship. More specifically, Gerdner outlined a specific theoretical framework in order to formalize and refine an individualized music listening for patients with dementia through the “*Mid-range theory of individualized music intervention for agitation*” (IMIA) [17]. The first factor on which IMIA is based concerns the perception of music by the person with dementia. Although the pathology may drastically reduce the ability to understand and produce language, the receptive and expressive skills concerning music are generally preserved much longer and beyond the severity of cognitive decline. For this reason, although the literature has not yet come to a univocal and solid explanation, we tend to consider music processing as partially independent from cognitive efficiency [18]. The second factor concerns the ability of music to elicit memories. As a powerful means of reminiscence, music can produce both pleasant and unpleasant memories, depending on the type of evoked stimuli, images and sensations linked to the person’s private experience [19]. In order to avoid the possibility that music may elicit negative memories, it must be selected (i.e., an “*individualized approach*”). It has to be part of the patient’s positive experience and should be based on his/her personal preferences (for example, popular music at the time of patient’s adulthood, or songs offered during religious or other services followed, etc.). As specified by Gerdner [16], the assessment must cover individual songs as well as preferred instruments and genres; if cognitive impairment affects the ability of the person to select music, it is possible to interview the caregiver to find this information.

Given these characteristics, such a kind of protocol seems to be promising as a complementary strategy to pharmacological treatment for people with dementia living in institutional settings. Starting from this assumption, the aim of our study was to evaluate the effect of an MT intervention adopting Gerdner and colleagues’ protocol in reducing neuropsychiatric symptoms reported by dementia patients and in ameliorating the caregiver’s burden.

## 2. Materials and Methods

### 2.1. Participants

A randomized controlled trial (RCT) was conducted at the RSA *Monsignor Bicchierai* in the Istituto Auxologico (Milan, Italy). A total of 60 residents and associated caregivers were randomly assigned to the Experimental Group (EG) (*n* = 30) and to the Control Group (CG) (*n* = 30). The residents underwent a complete psychogeriatric and neurological examination at the Institute, including the administration of the Mini Mental State Examination (MMSE). Inclusion criteria to the study for residents encompassed: (i) a diagnosis of dementia, according to the Diagnostic and Statistical Manual of Mental Disorders, Fourth Edition; (ii) age over 80 years; (iii) an MMSE score < 20, ranging from moderate to severe dementia [20]. The residents were excluded if they report: (i) a severe psychiatric condition; (ii) a hearing impairment; (iii) any other inability that may interfere in attending a 20-minute MT intervention; (iv) absence of a reliable informant caregiver. No restriction was applied for residents-associated caregivers. Eligible participants and their caregivers were provided with a detailed explanation of the study. All the patients signed an informed consent and for those with a severe cognitive deterioration, the consent was provided by the caregivers who were reassured of confidentiality and anonymity of the data collected during the study. Participants could withdraw from the study at any time without any effect on their usual care at the Facility.

### 2.2. Clinical Measures and Outcomes

Caregivers were administered the Caregiver Burden Inventory (CBI) [21] by a trained clinical psychologist dedicated to elderly care in the Facility. In addition, the Beck Depression Inventory-II [22] and the Penn State Worry Questionnaire [23] were used prior to the intervention. The caregivers were also interviewed about associated residents’ neuropsychiatric symptoms by the Neuropsychiatric Inventory (NPI) (Cummings et al., 1994) [7] reporting two main scores (NPI_aXb = frequency for severity; NPI_distress = caregiver’s distress). The effectiveness of the MT was expected as an improvement in the following outcome measures after the intervention: CBI total score, NPI_aXb and NPI_distress scores.

### 2.3. MT Intervention

**Figure 1:**
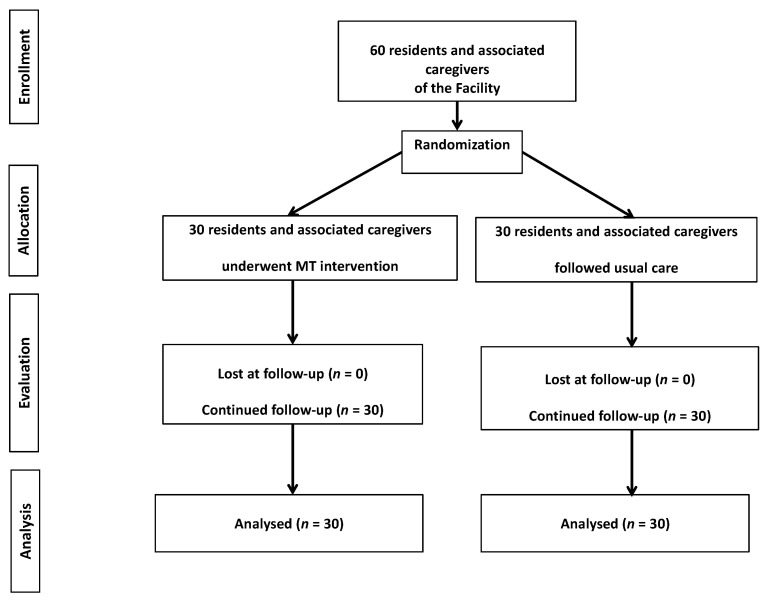
The study flow chart.

The residents and associated caregivers were allocated to the EG and the CG using a predetermined list of randomization, with 1:1 allocation ratio and they were blinded towards the intervention (Figure 1). All the participants completed the study protocol. In both cases, caregivers were considered part of the Facility staff signing the *Individualized Care Plan* designed by the multidisciplinary group (i.e., geriatrician, nursing coordinator, educator, social assistant, and clinical psychologist) for each resident and agreed to attend the Facility activities program during the intervention. While residents and associated caregivers of the CG followed the usual care provided by the Assisted Healthcare Residence staff (i.e., educational support and entertainment activities), residents and associated caregivers of the EG underwent an intervention of music listening strictly respecting Gerdner and colleagues’ protocol [24], as follows: (1) music selection according to patient’s preference by caregivers [25]; (2) music material file (i.e., Mp3) preparation for each resident, as a result of the collaboration between caregiver and psychologist; (3) MT intervention on residents’ room at the Facility as a quiet and comfortable environment (i.e., 30 min 2 times a week for 8 weeks, for a total of 16 sessions); (4) information provided to caregivers by the psychologist on patient’s monitoring during sessions (in case of agitation, music listening was interrupted).

### 2.4. Statistical Analysis

The collected data passed the Shapiro–Wilk test for normality distribution and Levene test for variances homogeneity. The comparability of the two study groups was first determined using T-tests for independent samples for continuous variables. Then, changes between groups after the intervention were compared by a mixed model ANCOVA by controlling for significant differences that resulted after the T-tests at baseline (Dependent variables: CBI; NPI_aXb; NPI distress; Factors: Time and Groups, EG vs. CG; Covariates: BDI; PSWQ). The effect size was calculated by the eta squared.

## 3. Results

### 3.1. Descriptive Analysis of the Whole Sample

Age and education of the residents were 89.50(±6.96) and 9.68(±5.20) years, respectively, 41.7% male and 58.3% female, with an MMSE of 9.45 ± 6.66. Age and education of the caregivers were of 61.7(±7.67) and of 11.5(±7.66) years, respectively. Descriptive statistics of clinical measures are shown in Table 1.

### 3.2. Comparison of the EG and the CG

The T-tests for independent samples revealed that groups did not differ in terms of CBI (t(58) = 1.019, *p* = 0.313), NPI_aXb (t(58) = 1.715, *p* = 0.490) and NPI_distress (t(58) = 0.025, *p* = 0.156) dimensions at baseline. Conversely, significant differences were found in terms of depression severity (BDI-II) (t(58) = 3.768, *p* = 0.044), and worry (PSWQ) (t(58) = 0.678, *p* = 0.009).

### 3.3. CBI Results

**Figure 2:**
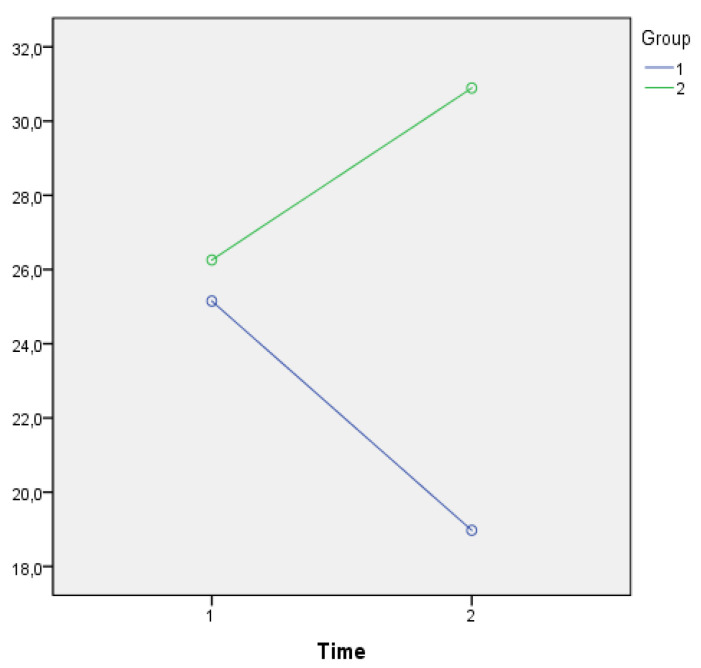
Comparison of the average CBI global scores in the EG (purple line) and in the CG over time (green line).

As shown in Figure 2, a Time*Group effect (λ = 0.872; F(1,56) = 8.038; *p* = 0.006; η^2^ = 0.128) was found with regard to CBI that decreases after the intervention in the EG while this trend was not shown for the CG.

### 3.4. NPI Results

**Figure 3:**
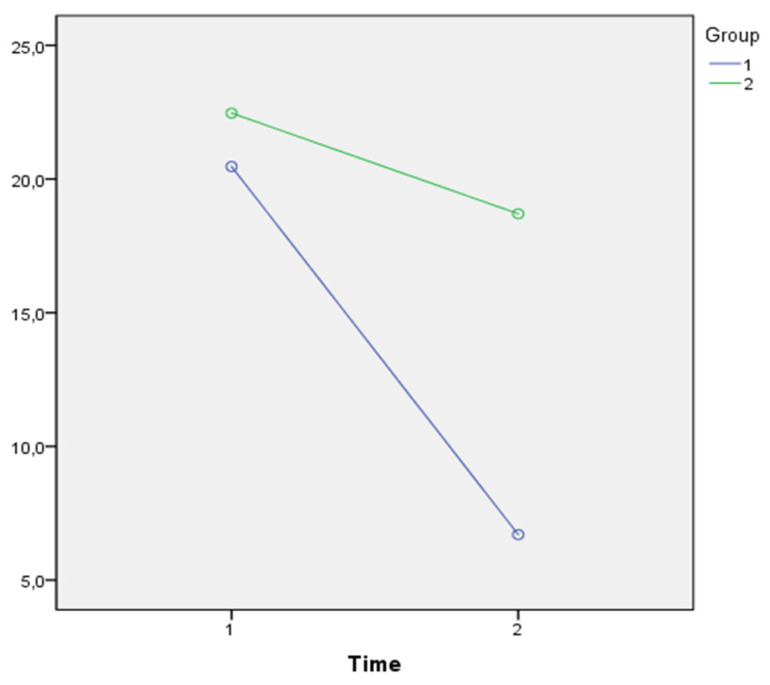
Comparison of the average NPI_aXb in the EG (purple line) and in the CG (green line) over time.

**Figure 4:**
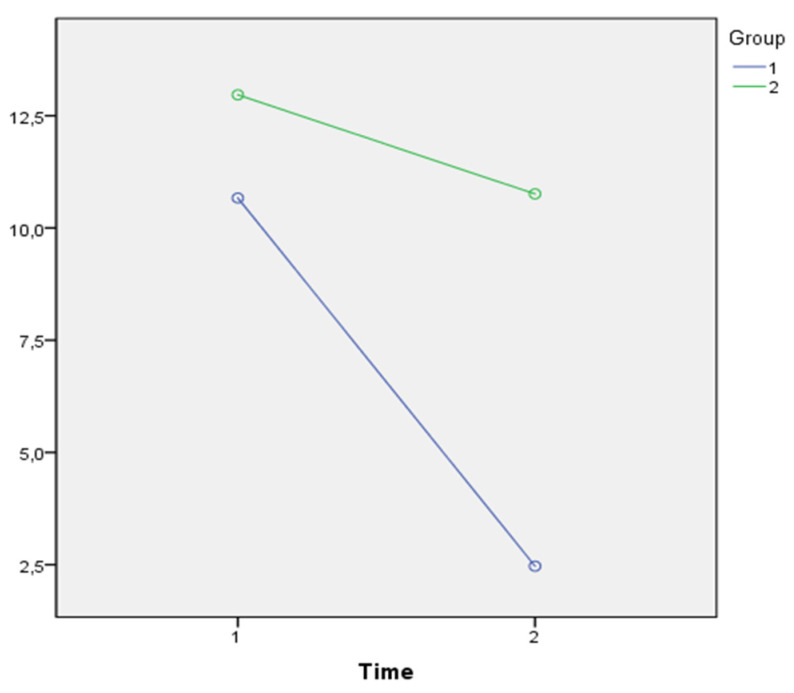
Comparison of the average NPI_distress in the EG (purple line) and in the CG over time (green line).

As shown in Figure 3, a Time*Group effect (λ = 0.740; F(1,56) = 20.343, *p* = 0.001; η^2^ = 0.260) was also found with regard to NPI_aXb, with a greater effect for the EG. Likewise, as shown in Figure 4, a Time*Group effect (λ = 0.779; F(1,56) = 16,165, *p* = 0.001; η^2^ = 0.221) was found with regard to NPI_distress, with a greater effect for the EG.

## 4. Discussion

We demonstrated that a structured MT intervention (i.e., 30 min two times a week for 8 weeks) based on Gerdner and colleagues’ protocol [24] ameliorates caregivers’ burden and reduces neuropsychiatric symptoms reported in assisted elderly residents with dementia better than usual care, both for their frequency/severity and perceived distress by caregivers. According to a recent 12-year longitudinal cohort study [26], understanding the natural course of neuropsychiatric symptoms in dementia is important for patient care planning and trial design. Remarkably, starting from a previous systematic literature review [27] highlighting how depression, agitation/aggression and apathy are the most distressing symptoms for caregivers assisting people with dementia, the MT intervention adopted reported an effect on neuropsychiatric symptoms as a whole, suggesting how it may be beneficial for a large spectrum of dimensions potentially impacting on patients’ behavior and caregivers’ health.

Other investigations have already shown a reduction in some neuropsychiatric symptoms associated with dementia after MT interventions. In detail, Garland et al. [28] showed that both listening to audiotapes with a conversation about positive experiences from the past and the exposure to a selection of songs that the individual used to enjoy in their youth are effective in reducing agitation. Holmes et al. [29] revealed that live interactive music is more effective than pre-recorded music in reducing apathy in moderate and severe dementia. Moreover, a case–control study [30] concluded that MT sessions consisting of singing songs chosen by the group accompanied by instruments significantly reduce agitation and anxiety in a sample of people suffering from moderate-to-severe Alzheimer’s dementia. More recently, Raglio and colleagues [31] completed a Randomized Controlled Trial (RCT) reporting that consecutive cycles of 12 active MT sessions three times a week is sufficient for observing a significant reduction in behavioral disorders in severely impaired patients with dementia. Finally, Sung et al. [32] investigated the effects of group music sessions of 30 min, twice a week for 6 weeks in institutionalized elders with dementia (i.e., five-minute warm-up session with movements and breathing; 20-minute session of active participation using percussion instruments; five minutes of soft music listening) founding that such a type of intervention is effective for anxiety reduction. Our study added a few thoughts on MT protocols highlighting the potential role of music in evoking emotional response associated with personal memories (i.e., autobiographical events) thanks to an individualized approach able to bypass cognitive impairment severity.

Further, our findings are in line with the latest published Cochrane review [33] reporting that providing people with dementia with at least five sessions of a music-based therapeutic intervention improves overall behavioral and psychological problems at the end of treatment. According to the guidelines of the *Italian Psychogeriatric Association* [34] highlighting the necessity to produce RCTs based on structured evidence-based music protocols for people with dementia, we would stress that Gerdner and colleagues’ schema represents an effective way to improve wellbeing both for people with dementia living in institutional settings and for their caregivers. Gerdner and colleagues’ protocol for the usage of personal music materials to evoke past memories of the patients may represent an original application of personalized medicine in dementia, even if more efforts are necessary to meet the clinical complexity of the disease and to build stronger evidence able to address rehabilitation practice.

However, our study had some limitations. In order to reach a better generalizability of results, larger randomized *double-blind* controlled trials with follow-up measuring maintenance effects are encouraged in the future. Indeed, interventions based on listening to the music usually present the greatest effect at the end of the intervention, without maintenance effect [35]. It is also necessary to develop clinical trials aiming to design standardized protocols depending on etiology and stage of dementia so they can be applied alongside psychological intervention (e.g., cognitive-behavioral therapy) or pharmacological treatment. In addition, the CBI includes items referred to daily living and it does not fulfil criteria to specifically evaluate residents at institutional settings. In order to implement future RCTs, researchers should also assume measures such as the Revised Scale for Caregiving Self Efficacy [36] with the scope of facilitating the development of improved caregiver strategies for dealing with stressors form care. Potential effects of medication received by patients with dementia that may influence results were also not taken into account.

## 5. Conclusions

We documented that a structured MT intervention administered for 8 weeks (20 min a day) in a relaxing way for patients with moderate-to-severe dementia living in institutional settings is able to reduce BPSD and ameliorate caregivers’ burden. Such an intervention was brief, safe, low-cost and can be replicated in similar contexts, without spending in excessive sanitary and human resources. A caregiver’s efficacy for managing BPSD is an important determinant of familiar stress and plays a pivotal role with regard to patients’ management. Implementing MT interventions with a more comprehensive assessment of caregivers’ profile may be advantageous in supporting institutionalized elderly people with dementia.

## Figures and Tables

**Table 1:** Clinical measures of the EG and the CG prior and after the intervention.

	EG (*n* = 30)	CG (*n* = 30)
BDI-II	9.23 ± 1.68	6.15 ± 1.12
PSWQ	49.80 ± 12.44	40.10 ± 15.39
CBI (baseline)	27.26 ± 13.37	24.06 ± 10.51
CBI (follow-up)	19.53 ± 10.40	30.53 ± 11.69
NPI_aXb (baseline)	20.46 ± 9.00	22.46 ± 12.96
NPI_aXb (follow-up)	6.70 ± 5.17	18.70 ± 8.65
NPI_distress (baseline)	10.66 ± 6.07	12.96 ± 6.21
NPI_distress (follow-up)	2.46 ± 2.06	10.66 ± 5.31

Data are expressed as mean ± standard deviation; EG: Experimental Groups; CG: Control Group; BDI-II: Beck Depression Inventory-II; PSWQ: Penn State Worry Questionnaire; CBI: Caregiver Burden Inventory; NPI_aXb: Neuropsychiatric Inventory_frequency for serverity; NPI_distress: Neuropsychiatric Inventory_distress.

## Data Availability

Details regarding data supporting results can be found at: http://tesionline.unicatt.it/handle/10280/59475, Accessed date: 11 April 2021.
